# Markers of Autonomic Function and Cancer Mortality Risk in Healthy Adults

**DOI:** 10.1016/j.jacadv.2026.103242

**Published:** 2026-09-18

**Authors:** Clare Meernik, David Leonard, Kerem Shuval, Carolyn E. Barlow, Andjelka Pavlovic, Samira Kamrudin, Lee W. Jones, Kelley Pettee Gabriel, Jason R. Carter, Nina Radford, Jarett D. Berry, Laura F. DeFina, Susan C. Gilchrist

**Affiliations:** a Kenneth H. Cooper Institute at Texas Tech University Health Sciences Center, Dallas, Texas, USA; b Julia Jones Matthews School of Population and Public Health, Texas Tech University Health Sciences Center, Lubbock, Texas, USA; c Department of Population Sciences, Beckman Research Institute of City of Hope, Duarte, California, USA; d Department of Epidemiology, University of Alabama at Birmingham, Birmingham, Alabama, USA; e Robbins College of Health and Human Sciences, Baylor University, Waco, Texas, USA; f Cooper Clinic, Dallas, Texas, USA; g University of Texas at Tyler School of Medicine, Tyler, Texas, USA; h Department of Medicine, University of North Carolina at Chapel Hill School of Medicine, Chapel Hill, North Carolina, USA

**Keywords:** autonomic function, cancer mortality, cardiorespiratory fitness

## Abstract

**Background:**

Dysfunction of the autonomic nervous system increases cardiovascular risk, but its contribution to cancer outcomes is less well-characterized.

**Objectives:**

The aim of this study was to evaluate associations between physiological measures reflective of autonomic function and all-site and site-specific cancer mortality in healthy adults.

**Methods:**

The study included 75,308 participants (26.8% women) in the Cooper Center Longitudinal Study who completed a preventive medicine examination with a treadmill test between 1971 and 2018. Gompertz mortality models estimated associations between autonomic markers—resting heart rate, heart rate reserve, systolic blood pressure response to exercise, and heart rate recovery—and cancer mortality through 2019. Analyses were sex-stratified when significant interactions were present. Models adjusted for age, clinical and lifestyle factors, and cardiorespiratory fitness.

**Results:**

Over a median follow-up of 21.9 years, 3,855 cancer deaths occurred. Lower heart rate reserve (HR per 10% lower: 1.05; 95% CI: 1.02-1.07) and heart rate recovery at 5 minutes (HR per 10 beats/min lower: 1.06; 95% CI: 1.03-1.09) were associated with higher all-site cancer mortality. Sex-specific associations were observed for certain markers: A higher resting heart rate was related to higher cancer mortality in men (HR per 10 beats/min: 1.04; 95% CI: 1.01-1.08), but not in women, whereas systolic blood pressure response associations were stronger in women. Associations with site-specific cancer mortality were particularly pronounced for lung cancer.

**Conclusions:**

In apparently healthy adults, multiple noninvasive markers of autonomic function were associated with cancer mortality, independent of cardiorespiratory fitness.

The autonomic nervous system (ANS) regulates key cardiopulmonary functions, including heart rate, blood pressure, cardiac contractility, vascular tone, and pulmonary vascular resistance.^1^ Dysfunction of the ANS, characterized by an imbalance between sympathetic and parasympathetic activity,^2^ increases the risk of cardiovascular disease and death.^3^, ^4^, ^5^, ^6^, ^7^ The health implications of autonomic dysfunction may also extend to cancer outcomes, as several studies have reported associations between markers of autonomic function and cancer incidence and mortality.^8^, ^9^, ^10^, ^11^, ^12^ Heightened sympathetic nervous system activity, in particular, is hypothesized to increase the risk of cancer mortality through mechanisms including β-adrenergic signaling and disruption of the immune response.^8^^,^^13^

Despite evidence linking autonomic function to cancer mortality in healthy populations, important knowledge gaps remain. Most studies have focused on a single indicator—resting heart rate—in relation to cancer mortality;^8^, ^9^, ^10^, ^11^ it is unclear whether other physiological measures are similarly predictive or whether associations differ by cancer site. Finally, few studies have accounted for cardiorespiratory fitness (CRF), which may have confounded prior estimates given CRF’s influence on autonomic function and its inverse relationship with cancer mortality.^14^, ^15^, ^16^, ^17^

To address these gaps, the current study estimated cause-specific associations between several physiological markers that may reflect autonomic regulation and all-site and site-specific cancer mortality, with and without adjustment for CRF. The markers evaluated were resting heart rate, heart rate reserve, systolic blood pressure (SBP) response to exercise, and heart rate recovery after exercise.

## Methods

### Study population

The study included apparently healthy men and women in the Cooper Center Longitudinal Study (CCLS), a prospective cohort designed to study lifestyle factors and health outcomes.^18^ Individuals participating in the CCLS received a preventive medicine examination at the Cooper Clinic (Dallas, Texas) and provided informed consent for study participation. CCLS participants are predominantly White and have access to preventive health care.^19^ The CCLS received ethical approval from the Texas Tech University Health Sciences Center Institutional Review Board (IRB-FY2026-339).

Among 102,805 CCLS participants who completed a modified Balke treadmill test at their baseline clinic visit, we excluded the following participants: missing covariates, including body mass index (BMI) (n = 8,045), glucose (n = 3,771), cholesterol (n = 24), or SBP (n = 332); BMI <18.5 kg/m^2^ (underweight) (n = 1,082); clinic visit occurred in 2019 or later (less than 1 year of follow-up for mortality) (n = 8,178); died within 1 year of the clinic visit (n = 86); personal history of myocardial infarction (n = 823), stroke (n = 253), or cancer (n = 3,540); or missing resting heart rate, heart rate reserve, SBP response, or heart rate recovery at 5 minutes (n = 1,363). No participants were excluded based on treadmill test performance (eg, achieving <85% of age-predicted maximal heart rate), as this may reflect underlying autonomic dysfunction. The analytical sample included 75,308 participants.

Heart rate recovery at 1 and 2 minutes was inconsistently captured during early exam years; therefore, analyses of these markers were conducted in a subsample restricted to participants with available measurements (n = 43,022), hereafter referred to as the heart rate recovery at 1 and 2 minutes (HRR-1/2) subsample.

### Markers of autonomic function

Autonomic function can be evaluated using various approaches, including indirect, noninvasive measures derived from heart rate and blood pressure responses before, during, and after exercise.^20^ We examined 4 physiological markers that may reflect autonomic regulation: resting heart rate, percentage of heart rate reserve used at peak exercise (also referred to as chronotropic index), SBP response to exercise, and heart rate recovery after exercise.^20^, ^21^, ^22^

Resting heart rate and SBP were obtained during the clinic visit before the initiation of exercise. Resting heart rate was recorded via electrocardiogram (ECG) after a 5-minute rest in the supine position.^8^ Resting SBP was obtained using standard auscultatory methods after a minimum of 5 minutes seated rest.^8^ Data during exercise was obtained from a treadmill test using a modified Balke protocol.^23^^,^^24^ Upon completion of the exercise test—at volitional fatigue or medical termination—participants completed an active recovery of 5 minutes during which the treadmill speed and grade were set to 2.0 mph and 0%, respectively.^25^ Heart rate during exercise and in recovery was recorded via ECG.^25^

Heart rate reserve was calculated using the following formula: (maximal heart rate during exercise − resting heart rate) / (age-predicted maximal heart rate − resting heart rate) × 100. Age-predicted maximal heart rate was estimated as: 220 − age. SBP response to exercise was calculated as: maximal SBP − resting SBP. Heart rate recovery was calculated as maximal heart rate − heart rate at 1, 2, or 5 minutes after completion of the exercise test.

### Cancer mortality

Follow-up for cancer mortality extended through 2019. The underlying cause of death was identified from the National Death Index Plus. All-site cancer mortality was defined as death from malignant cancer (International Classification of Diseases-10th Revision [ICD-10] C00-C97). Site-specific cancer mortality included death from malignant lung (ICD-10 C33-34), pancreatic (ICD-10 C25), colorectal (ICD-10 C18-21), prostate (ICD-10 C61), or breast (ICD-10 C50) cancer.

### Covariates

All covariates were collected at the clinic visit, including age, clinic visit year, sex, BMI (kg/m^2^), CRF (metabolic equivalents), current smoking (yes/no), resting SBP (mm Hg), fasting glucose (mg/dL), and total cholesterol (mg/dL). To calculate BMI, weight and height were measured using a standard clinical scale and stadiometer, respectively.^26^ CRF (metabolic equivalents) was estimated based on the final treadmill speed and grade of a treadmill test using a modified Balke protocol;^23^^,^^24^^,^^27^ time on the treadmill is strongly correlated with directly measured VO_2max_.^28^^,^^29^ Resting systolic and diastolic blood pressure were assessed using standard auscultatory methods.^30^ Glucose and cholesterol were measured via blood samples after a 12-hour overnight fast.

### Statistical analysis

Descriptive characteristics of the sample are presented overall and by sex and vital status at the end of follow-up. Gompertz mortality models were used to estimate cause-specific HRs and 95% CIs for the associations between each marker of autonomic function and cancer mortality.^31^ The parametric mortality model allowed us to accommodate missing data on current smoking without imputation using the maximum likelihood method.^32^ Gompertz models assume hazard functions that increase exponentially with age, making them well-suited for modeling mortality.^33^^,^^34^ To ensure consistent directionality across markers, heart rate reserve and heart rate recovery were reverse-coded so that lower values reflected worse autonomic regulation. All-site and site-specific (lung, pancreatic, colorectal, prostate, and breast) cancer mortality was assessed for each marker of autonomic function; site-specific cancer mortality was not evaluated in the HRR-1/2 subsample because of limited events. Markers of autonomic function were analyzed as continuous exposures in separate models; linearity of each exposure was confirmed by examining Martingale residual plots.

Models were adjusted for clinic visit age, year, sex, BMI, current smoking status, resting SBP, fasting glucose, and total cholesterol, with or without additional adjustment for CRF. Quadratic terms for age and year were included to improve model fit. Models were sex-stratified when significant interactions were present. All analyses were programmed in SAS/STAT version 9.4 (SAS Institute, Inc). A 2-tailed *P* value <0.05 was considered statistically significant.

### Sensitivity analysis

Three sensitivity analyses were conducted. First, to account for potential reverse causality, we excluded participants with less than 5 years of follow-up, including those who died within 5 years of the clinic visit. Second, we excluded participants with a personal history of hypertension, who may have been prescribed blood pressure medications that affect heart rate, and those with an abnormal or equivocal resting ECG. Third, we assessed the relationship between each marker of autonomic function and all-cause mortality to assess whether cancer-specific associations were accompanied by corresponding differences in overall mortality.

## Results

The analytical sample included 75,308 participants (26.8% women) who had a clinic visit between 1971 and 2018 at a mean age of 44.6 (SD: 9.9) years (Table 1). During a median follow-up of 21.9 years (interquartile range: 13.8-33.5 years), 3,855 cancer deaths were identified (3,211 among men; 644 among women). Participants who died of cancer were older at the clinic visit and had a less-favorable cardiovascular risk profile, including a higher prevalence of smoking, lower CRF, and higher glucose, cholesterol, and blood pressure. The number of deaths and mortality rates for all-site and site-specific cancer mortality are presented in Table 2.

**Table 1:** Characteristics at Clinic Visit (1971-2018) Among Study Participants in the Cooper Center Longitudinal Study (N = 75,308), Overall and by Sex and Vital Status at the End of Follow-up

	Overall (N = 75,308)	Men (n = 55,091)		Women (n = 20,217)	
		Cancer Death (n = 3,211)	No Cancer Death (n = 51,880)	Cancer Death (n = 644)	No Cancer Death (n = 19,573)
Characteristic					
Age (y)	44.6 (9.9)	48.5 (9.4)	44.3 (9.6)	49.8 (10.3)	44.8 (10.5)
Body mass index (kg/m^2^)	26.3 (4.4)	26.8 (3.7)	27.1 (4.1)	24.2 (4.5)	24.1 (4.6)
Current smoking^a^, n (%)	11,195 (17.9)	877 (36.9)	8,683 (20.2)	104 (23.5)	1,531 (9.1)
Cardiorespiratory fitness (METs)	10.9 (2.5)	10.4 (2.3)	11.5 (2.4)	8.5 (2.0)	9.5 (2.1)
Fasting glucose (mg/dL)	98.3 (17.0)	102.9 (17.6)	100.0 (17.6)	97.3 (21.7)	93.2 (13.5)
Total cholesterol (mg/dL)	204.0 (39.8)	217.0 (41.0)	205.2 (40.2)	209.3 (40.8)	198.6 (37.8)
Resting systolic blood pressure (mm Hg)	119.7 (14.1)	124.0 (14.6)	121.9 (13.2)	116.9 (15.9)	113.3 (14.3)
Resting diastolic blood pressure (mm Hg)	80.1 (9.9)	82.4 (9.8)	81.5 (9.6)	77.2 (9.8)	76.1 (9.5)
Resting heart rate (beats/min)	62.2 (10.7)	62.6 (10.9)	61.2 (10.7)	65.1 (10.8)	64.8 (10.5)
Heart rate reserve (%)^b^	101.1 (12.4)	101.4 (14.4)	101.5 (12.3)	99.8 (12.5)	100.1 (12.3)
Systolic blood pressure response to exercise (mm Hg)^c^	64.6 (21.7)	65.3 (21.7)	69.3 (20.8)	51.8 (21.1)	52.4 (19.1)
Heart rate recovery at 5 min post exercise (beats/min)^d^	66.2 (15.2)	67.8 (15.0)	66.7 (15.2)	66.9 (16.1)	64.9 (15.2)

Values are mean (SD).

MET = metabolic equivalent.

a Percentages exclude missing observations. Current smoking was missing for 17.6% of men and 15.0% of women. Observations with missing smoking status were included in multivariable models using the maximum likelihood method.

b Heart rate reserve was calculated as: (maximal heart rate during exercise − resting heart rate)/(age-predicted maximal heart rate − resting heart rate) × 100.

c Systolic blood pressure (SBP) response to exercise was calculated as: maximal SBP − resting SBP.

d Heart rate recovery at 5 min was calculated as: maximal heart rate − heart rate at 5 min post exercise.

**Table 2:** Number of Deaths and Mortality Rates for All-Site and Site-Specific Cancer Mortality Among Men and Women in the Cooper Center Longitudinal Study (N = 75,308)

	Overall (N = 75,308)		Men (n = 55,091)		Women (n = 20,217)	
	Deaths	Deaths per 1,000 Person-Years	Deaths	Deaths per 1,000 Person-Years	Deaths	Deaths per 1,000 Person-Years
All-site	3,855	2.20	3,211	2.43	644	1.51
Lung	687	0.39	589	0.45	98	0.23
Prostate (men)	389	0.29	389	0.29	—	—
Breast (women)	113	0.27	—	—	113	0.27
Pancreas	375	0.21	315	0.24	60	0.14
Colorectal	273	0.16	230	0.17	43	0.10

The HRR-1/2 subsample included 43,022 participants (31.8% women) who had a clinic visit between 1975 and 2018 at a mean age of 46.1 (SD: 9.6) years (Supplemental Table 1). During a median follow-up of 15.9 years (IQR: 9.8-20.6 years), 722 cancer deaths were identified (532 among men; 190 among women). Similar to the overall sample, participants who died of cancer had a less-favorable cardiovascular risk profile. Furthermore, compared with participants in the overall sample who died of cancer, participants in the HRR-1/2 subsample who died of cancer were older at the clinic visit, had a higher BMI, and were less likely to currently smoke.

### Resting heart rate

A statistically significant sex interaction was observed for the association between resting heart rate and all-site cancer mortality (*P* = 0.037). Among men, each 10-beats/min higher resting heart rate was associated with a 9% (95% CI: 5%-12%) higher risk of all-site cancer mortality in an adjusted model without CRF (Figure 1). After adjustment for CRF, the association remained, though slightly attenuated (HR: 1.04; 95% CI: 1.01-1.08). For site-specific cancer mortality, positive associations were observed for lung (HR: 1.19; 95% CI: 1.11-1.29) and pancreatic cancer (HR: 1.16; 95% CI: 1.05-1.29), which were attenuated after CRF adjustment.

**Figure 1:**
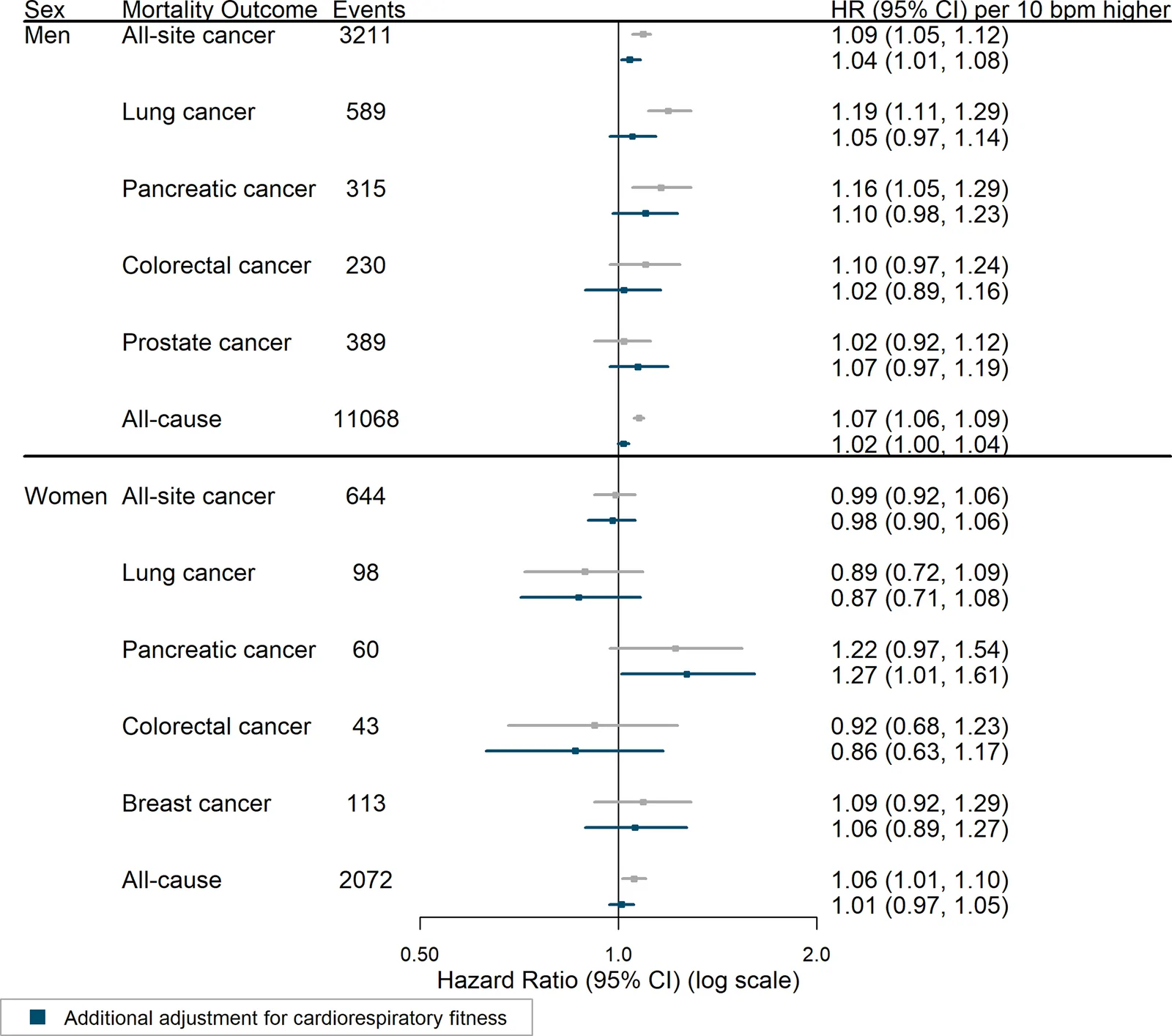
Sex-Specific Associations Between Resting Heart Rate and Mortality Hazard ratios for all-site cancer mortality, site-specific cancer mortality, and all-cause mortality are adjusted for clinic visit age, exam year, body mass index, smoking status, resting systolic blood pressure, fasting glucose, and total cholesterol, with or without additional adjustment for cardiorespiratory fitness (CRF). A statistically significant sex interaction was observed for all-site cancer mortality (*P* = 0.037). Among men (n = 55,091), a higher resting heart rate was associated with an increased risk of death from any cancer, independent of CRF. Positive associations with lung and pancreatic cancers were attenuated after adjustment for CRF. Among women (n = 20,217), a higher resting heart rate was associated with pancreatic cancer mortality only, independent of CRF.

Among women, resting heart rate was not associated with all-site cancer mortality (Figure 1). A positive association with pancreatic cancer mortality was observed only in the fully adjusted model including CRF (HR: 1.27; 95% CI: 1.01-1.61).

### Heart rate reserve

No statistically significant sex interaction was observed for the association between heart rate reserve and all-site cancer mortality (*P* = 0.807). Among men and women, each 10% lower heart rate reserve during peak exercise was associated with a 7% (95% CI: 4%-9%) higher risk of all-site cancer mortality in an adjusted model without CRF (Figure 2). The association remained similar after further adjustment for CRF (HR: 1.05; 95% CI: 1.02-1.07). For site-specific cancer mortality, positive associations were observed for lung (HR: 1.17; 95% CI: 1.11-1.23), pancreatic (HR: 1.10; 95% CI: 1.02-1.19), and colorectal cancer (HR: 1.13; 95% CI: 1.04-1.23), which were attenuated after CRF adjustment. Only the association with lung cancer mortality remained statistically significant (HR: 1.07; 95% CI: 1.02-1.14).

**Figure 2:**
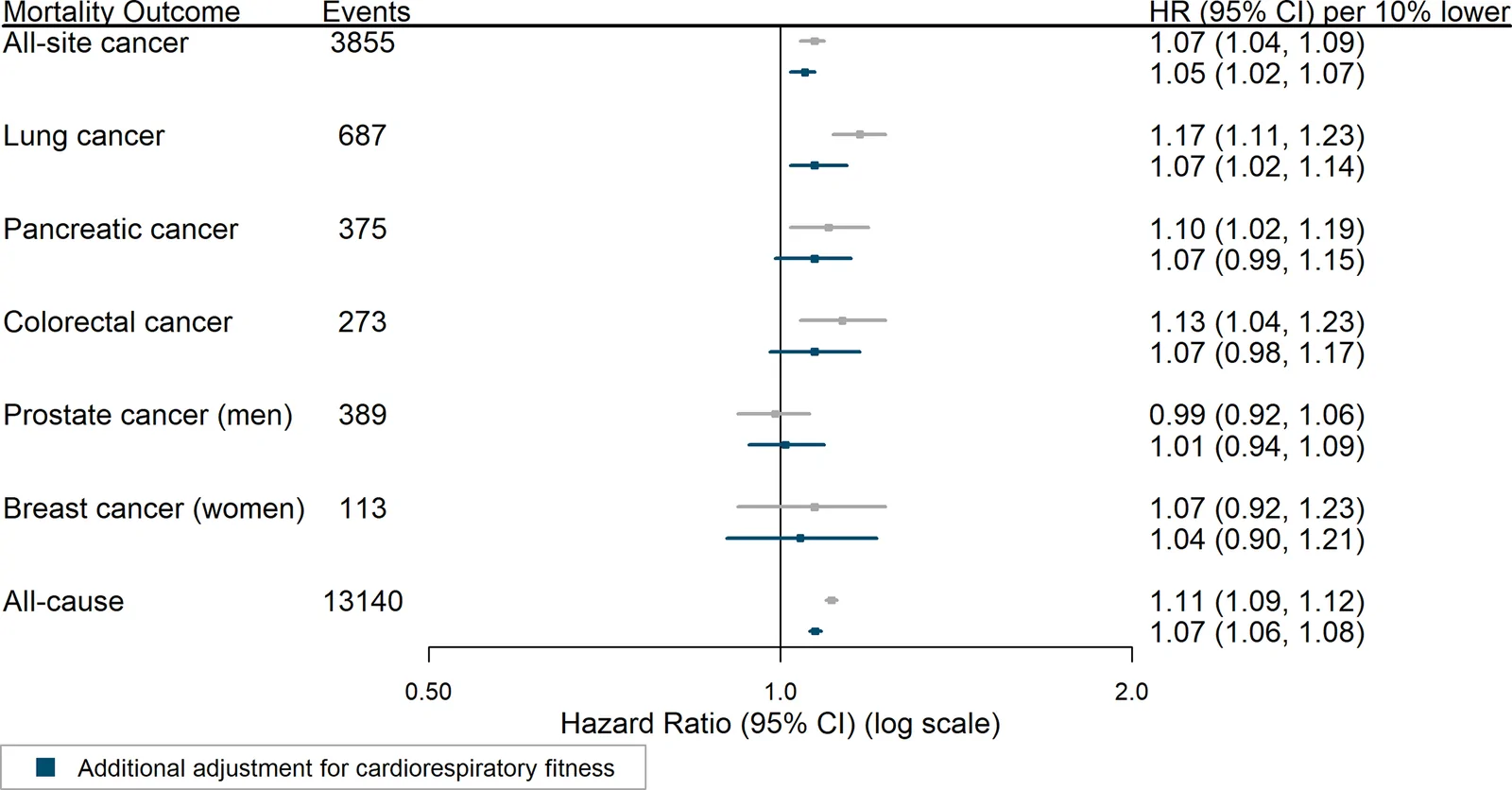
Associations Between Heart Rate Reserve and Mortality Hazard ratios for all-site cancer mortality, site-specific cancer mortality, and all-cause mortality are adjusted for clinic visit age, exam year, sex, body mass index, smoking status, resting systolic blood pressure, fasting glucose, and total cholesterol, with or without additional adjustment for cardiorespiratory fitness (CRF) (n = 75,308). Lower heart rate reserve was associated with an increased risk of death from any cancer, as well as lung cancer, independent of CRF. Positive associations with pancreatic and colorectal cancers were attenuated after adjustment for CRF.

### SBP response to exercise

A statistically significant sex interaction was observed for the association between SBP response and all-site cancer mortality (*P* = 0.020). Among men, each 10 mm Hg higher SBP response was associated with a 2% (95% CI: 0%-4%) higher risk of all-site cancer mortality in an adjusted model with CRF (Figure 3). A positive association with lung cancer mortality was also observed in a fully adjusted model with CRF (HR: 1.05; 95% CI: 1.01-1.10). No other site-specific associations were significant.

**Figure 3:**
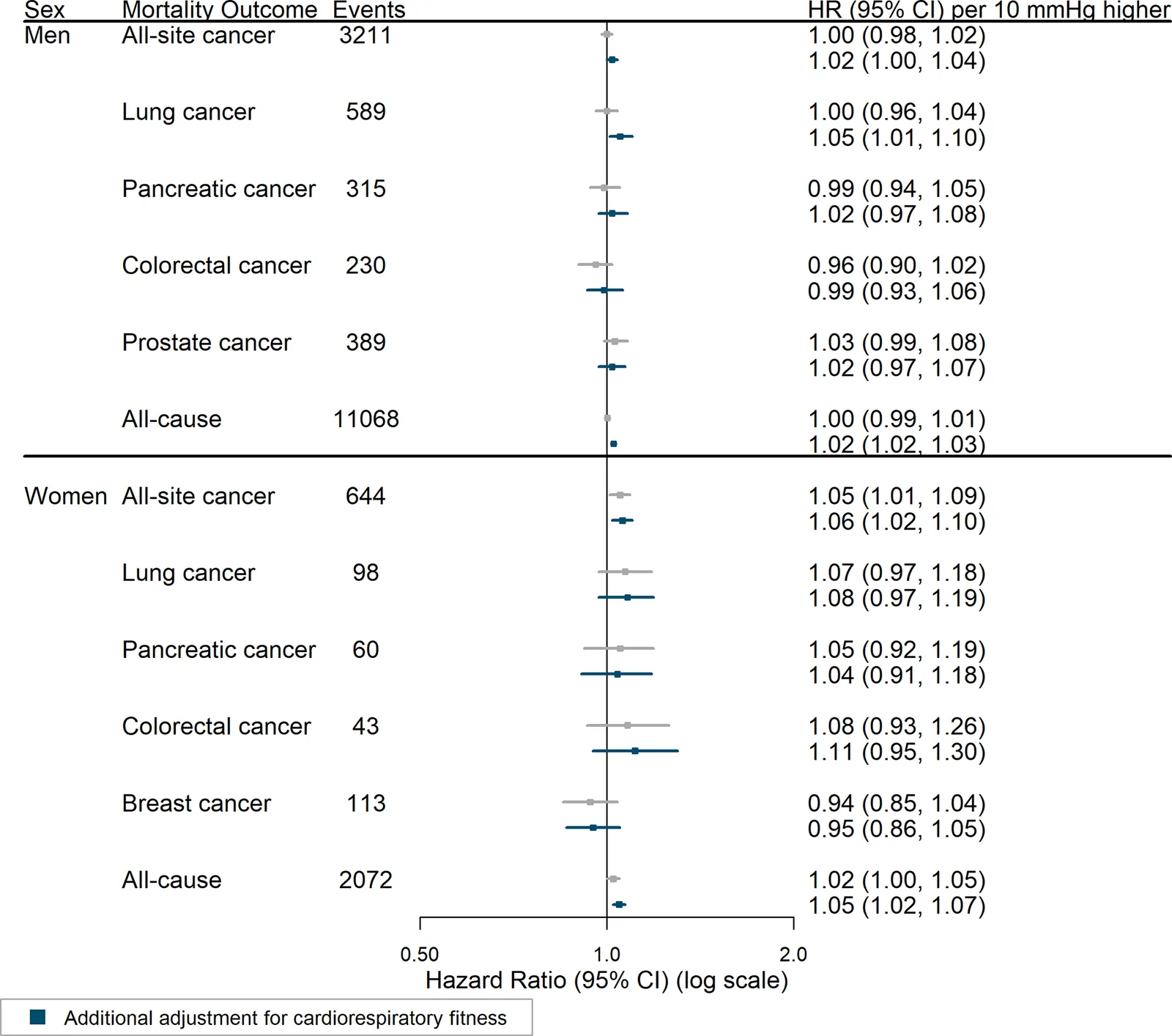
Sex-Specific Associations Between Systolic Blood Pressure Response to Exercise and Mortality Hazard ratios for all-site cancer mortality, site-specific cancer mortality, and all-cause mortality are adjusted for clinic visit age, exam year, body mass index, smoking status, resting systolic blood pressure (SBP), fasting glucose, and total cholesterol, with or without additional adjustment for cardiorespiratory fitness (CRF). A statistically significant sex interaction was observed for all-site cancer mortality (*P* = 0.020). Among men (n = 55,091), a higher SBP response was associated with an increased risk of death from any cancer, as well as lung cancer, independent of CRF. Among women (n = 20,217), a higher SBP response was associated with an increased risk of death from any cancer, independent of CRF.

Among women, each 10 mm Hg higher SBP response was associated with a 5% (95% CI: 1%-9%) higher risk of all-site cancer mortality in an adjusted model without CRF, which persisted after CRF adjustment (HR: 1.06; 95% CI: 1.02-1.10) (Figure 3). Positive associations with site-specific cancer mortality were observed for all cancers except breast cancer, although estimates were not statistically significant.

### Heart rate recovery after exercise

No statistically significant sex interaction was observed for the association between heart rate recovery at 5 minutes and all-site cancer mortality (*P* = 0.093). Among men and women, each 10 beats/min lower heart rate recovery at 5 minutes was associated with a 9% (95% CI: 6%-12%) higher risk of all-site cancer mortality in an adjusted model without CRF (Figure 4). The association persisted after CRF adjustment (HR: 1.06; 95% CI: 1.03-1.09). For site-specific cancer mortality, positive associations were observed for lung (HR: 1.19; 95% CI: 1.13-1.27) and pancreatic cancer (HR: 1.11; 95% CI: 1.02-1.20), which were attenuated after CRF adjustment; only the association with lung cancer remained statistically significant (HR: 1.07; 95% CI: 1.01-1.15).

**Figure 4:**
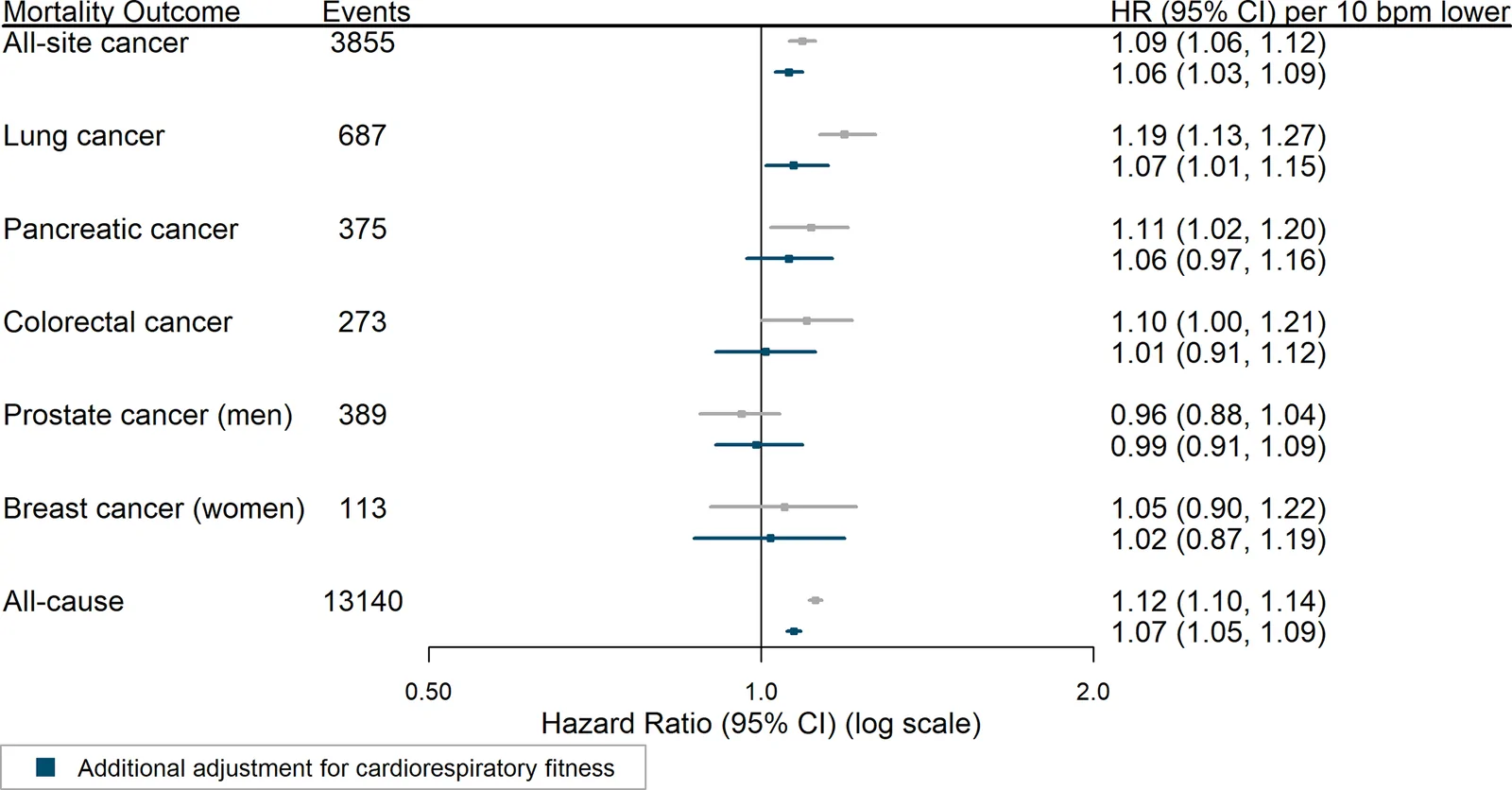
Associations Between Heart Rate Recovery at 5 Minutes and Mortality Hazard ratios for all-site cancer mortality, site-specific cancer mortality, and all-cause mortality are adjusted for clinic visit age, exam year, sex, body mass index, smoking status, resting systolic blood pressure, fasting glucose, and total cholesterol, with or without additional adjustment for cardiorespiratory fitness (CRF) (n = 75,308). Lower heart rate recovery at 5 minutes was associated with an increased risk of death from any cancer, as well as lung cancer, independent of CRF. A positive association with pancreatic cancer was attenuated after adjustment for CRF.

In the HRR-1/2 subsample, no statistically significant sex interaction was observed for heart rate recovery at 1 minute (*P* = 0.412) or 2 minutes (*P* = 0.235). Among men and women, slower heart rate recovery at both time points was associated with a 14% higher risk of all-site cancer mortality in adjusted models without CRF, which remained significant after CRF adjustment (HR per 10 beats/min lower recovery at 1 minute: 1.11; 95% CI: 1.01-1.21; HR per 10 beats/min lower recovery at 2 minutes: 1.09; 95% CI: 1.02-1.18) (Supplemental Table 2).

### Sensitivity analysis

Estimates remained similar in sensitivity analysis excluding participants with less than 5 years of follow-up (n = 4,909) (Supplemental Table 3) and in separate analysis excluding participants with a personal history of hypertension (n = 11,662) and those with an abnormal or equivocal resting ECG (n = 2,984) (Supplemental Table 4). Furthermore, associations between each marker of autonomic function and all-cause mortality were consistent with those observed for cancer mortality, suggesting that the cancer-specific findings were accompanied by corresponding differences in overall mortality (Figure 1, Figure 2, Figure 3, Figure 4).

## Discussion

This cohort study of more than 75,000 men and women provides novel evidence that several noninvasive physiological measures reflective of autonomic function—extending beyond resting heart rate—were related to cancer mortality risk in a healthy population, independent of CRF. Specifically, a higher resting heart rate, lower heart rate reserve, elevated SBP response to exercise, and slower heart rate recovery after exercise were each associated with an increased risk of all-site and site-specific cancer mortality (Central Illustration). By including site-specific cancer outcomes and additional autonomic markers, these findings expand opportunities for clinical risk prediction and inform investigation of the mechanisms that may link autonomic function to cancer outcomes.

**Central Illustration:**
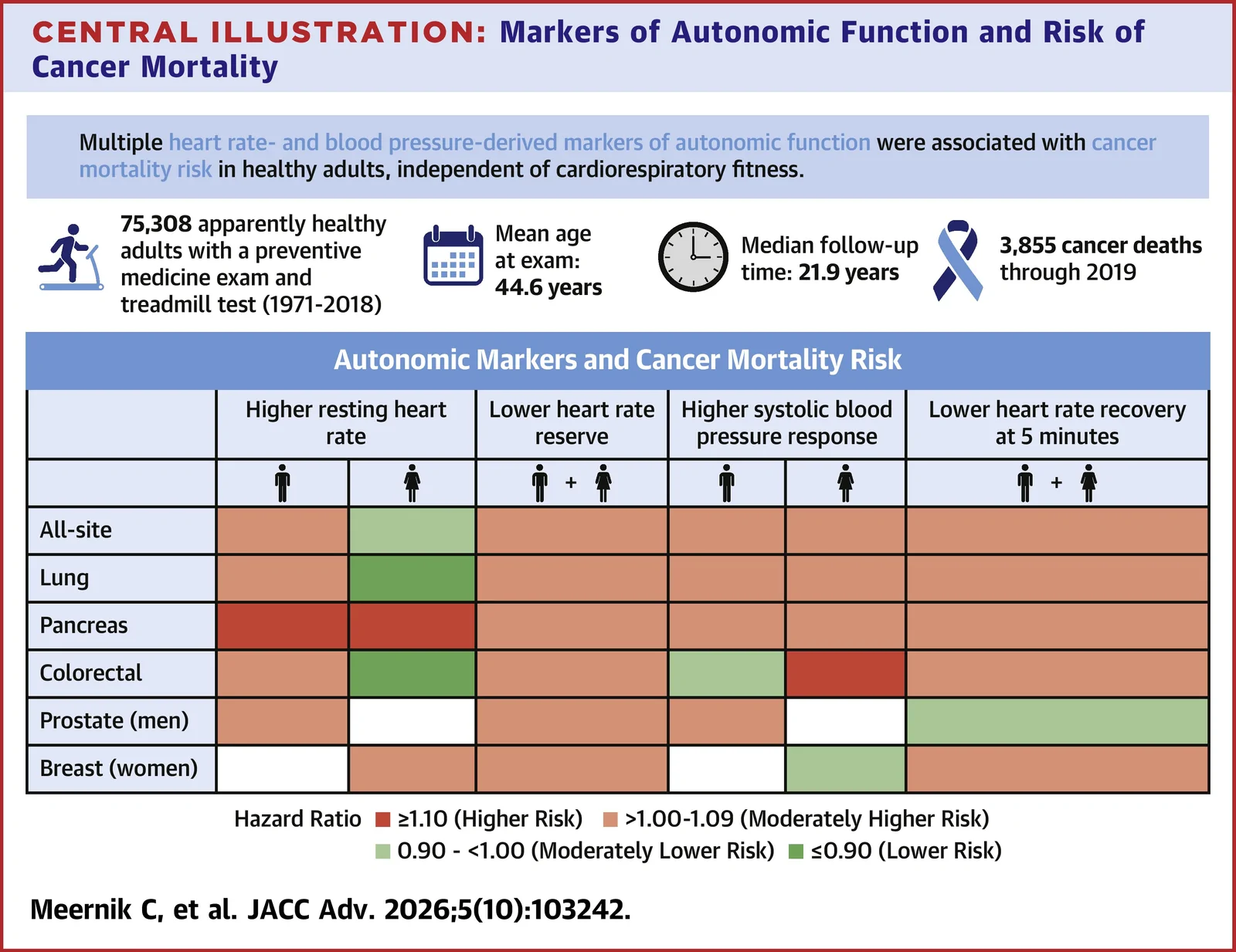
Markers of Autonomic Function and Risk of Cancer Mortality In a large cohort of 75,308 apparently healthy men and women who underwent a preventive medicine examination and treadmill test in midlife between 1971 and 2018, a higher resting heart rate, lower heart rate reserve, higher systolic blood pressure response to exercise, and lower heart rate recovery at 5 minutes post exercise were associated with all-site and site-specific cancer mortality, independent of cardiorespiratory fitness.

Autonomic dysfunction predicts adverse cardiovascular outcomes and shorter survival in patients with cancer,^35^, ^36^, ^37^, ^38^ but its role prior to disease onset remains less well established. In this cohort of apparently healthy adults, several markers of autonomic function were associated with cancer mortality, with evidence of certain sex-specific patterns. Sex differences were observed in SBP response to exercise, with stronger positive associations with cancer mortality among women. Although previous studies have suggested that a *decreased* SBP response during exercise may reflect autonomic abnormalities,^22^^,^^39^ an exaggerated response could indicate impaired vascular function.^40^^,^^41^ These findings warrant further investigation, particularly related to sex differences in blood pressure regulation and the role of arterial stiffness on peak SBP.^41^ Sex differences were also observed for resting heart rate. Among men, a higher resting heart rate was associated with increased mortality from all cancers combined, as well as lung and pancreatic cancers. Among women, an association was observed only for pancreatic cancer mortality. These results are consistent with prior studies showing a positive relationship between resting heart rate and both all-site and site-specific cancer mortality in men,^10^^,^^12^ while findings among women have been inconsistent.^10^^,^^42^, ^43^, ^44^ Sex differences in autonomic regulation, including hormonal influences,^45^ may contribute to these patterns, but have not been well characterized and warrant further study.

Similar to the present study, a previous analysis in the CCLS reported a positive relationship between resting heart rate and all-site cancer mortality independent of CRF (men and women combined).^8^ The present study extends that analysis by including nearly 20 years of additional follow-up, nearly 3,000 additional cancer deaths, and evaluation of 3 additional markers of autonomic function. Findings demonstrate that—in addition to resting heart rate—heart rate reserve, SBP response, and heart rate recovery are also associated with cancer mortality independent of CRF.

Thus, while heart rate and blood pressure–related markers of autonomic function may reflect CRF level, several mechanisms have been proposed to explain their associations with cancer mortality. Markers suggestive of autonomic dysfunction may indicate increased sympathetic nervous system activity.^8^^,^^36^ This excessive activation can affect beta-adrenergic signaling pathways involved in processes such as inflammation and angiogenesis; dysregulate the hypothalamic-pituitary-adrenal axis; and disrupt the immune response, potentially contributing to cancer development and progression.^8^^,^^13^^,^^36^ In addition, autonomic dysfunction may influence cancer risk and survival indirectly through its adverse effects on cardiovascular and metabolic health,^6^^,^^46^ which share common risk factors and biological pathways with cancer.^47^^,^^48^ Site-specific biological mechanisms are particularly important to investigate, including for lung, pancreatic, colorectal, and breast cancer. Impaired autonomic function has been associated with poorer survival in patients with these cancers,^36^, ^37^, ^38^^,^^49^ but its role in cancer development and progression before diagnosis is less clear. Among the site-specific findings, the observed associations of resting heart rate, heart rate reserve, and heart rate recovery with pancreatic cancer mortality are particularly notable given the limited opportunities for early detection and the poor prognosis of this cancer.^50^

Distinguishing the relative contributions of these physiological measures of autonomic function as risk markers for cancer incidence vs cancer survival is critical to identify potential targets for intervention. In addition to evaluating the relationship between autonomic markers and site-specific cancer incidence, assessing the role of autonomic function in survival among cancer patients may clarify whether these markers are associated with a more advanced stage at diagnosis, reduced tolerance to cancer treatment, or represent broader physiological dysregulation that may indirectly influence cancer risk and mortality through shared risk factors (eg, lifestyle behaviors and comorbidities). The findings of the present study may reflect a combination of these pathways, including associations with both cancer incidence and survival, as well as competing health risks, rather than a single underlying mechanism.

Among the multiple markers of autonomic function evaluated in this study, heart rate recovery after exercise appeared to be particularly informative for cancer mortality risk prediction. Heart rate recovery reflects the balance between the 2 branches of the ANS and can be classified into 2 phases: a fast phase within the first minute after exercise cessation, and a slow phase thereafter.^51^ Parasympathetic reactivation is thought to influence recovery during the fast phase, whereas sympathetic withdrawal, along with continued parasympathetic reactivation, contributes more to recovery during the slow phase.^46^^,^^51^ Compared to resting heart rate and heart rate reserve, delayed heart rate recovery is a stronger predictor of *all-cause* mortality,^52^ but its evaluation in relation to cancer mortality remains limited and should be further investigated in diverse cohorts.

Measures of heart rate and blood pressure that reflect autonomic regulation have traditionally been obtained through exercise testing in clinical and research settings but are now increasingly accessible through consumer wearable devices. While the standardization, integration, and interpretation of wearable data in clinical practice remain challenging,^53^^,^^54^ more widely accessible data on physiological markers of autonomic function at an individual patient level present opportunities to enhance care across the cancer control continuum. For instance, in healthy populations, these metrics may improve cancer risk prediction and highlight the importance of behavior change and screening for higher-risk individuals. Meanwhile, in patients with cancer, these metrics could be incorporated into cancer care to offer additional prognostic information. Furthermore, as wearables become more prevalent and validated for reliability, individual-level changes in markers of autonomic function over time can be more readily captured and should be considered in relation to cancer outcomes.

### Study limitations

Limitations of this study should be considered. First, given the observational study design, physiological measures reflective of autonomic function should be interpreted as markers of cancer mortality risk rather than causal factors. Further investigation of the mechanisms linking autonomic function to cancer outcomes—distinguishing between cancer development and survival after cancer—may strengthen clinical risk prediction and inform potential targets for screening and intervention. Second, the majority of the study sample was White and sought preventive health care, which limits the generalizability to more racially and ethnically diverse populations, as well as to individuals with limited access to care. Future studies should evaluate whether these associations persist in more diverse and underserved populations. Third, markers of autonomic function were assessed before cancer diagnosis. Thus, we could not determine how changes in autonomic function after diagnosis (eg, due to the cancer itself or its treatment^55^^,^^56^) may have affected survival. Fourth, data on the use of medications that may affect heart rate were incomplete. However, findings were unchanged in the sensitivity analysis that excluded participants with a personal history of hypertension or an abnormal/equivocal resting ECG. Fifth, certain site-specific estimates in women were less precise and less well-powered. Accordingly, findings should be interpreted cautiously and used to inform future studies examining sex- and site-specific pathways linking autonomic function with cancer mortality. Lastly, autonomic function is complex and lacks a single gold-standard measure.^46^ Several established markers, such as heart rate variability and autonomic reflex testing,^35^^,^^46^^,^^55^ were not available in this study and should be considered in relation to cancer mortality in future research. The markers included here are noninvasive and readily obtained during a preventive medicine examination with exercise testing, but they represent indirect indicators of autonomic regulation. Data on additional markers could provide a more comprehensive characterization of autonomic function and improve risk stratification for cancer mortality.^35^^,^^57^

## Conclusions

This study is among the first to evaluate multiple physiological measures reflective of autonomic function in relation to all-site and site-specific cancer mortality in healthy adults. Nearly 4,000 cancer deaths were identified from the National Death Index, which allowed for site-specific analyses examining associations between cancer mortality and several markers of autonomic function beyond resting heart rate. The analysis accounted for multiple lifestyle and clinical factors, including CRF estimated via a treadmill test. The consistency of the positive associations across several markers, even after adjustment for CRF, suggests an independent role of both CRF and autonomic function in cancer mortality.

Findings from this large cohort may help identify healthy adults at elevated risk of cancer mortality using simple, noninvasive markers of autonomic function. These markers are routinely collected during exercise testing and will be increasingly available to patients through wearables.^53^^,^^54^ Once validated, data from wearables might provide real-time health metrics to the general population and may promote discussions regarding lifestyle modifications and cancer screening. Autonomic dysfunction may be a target for future cancer-prevention efforts and improved risk stratification.

Perspectives **COMPETENCY IN MEDICAL KNOWLEDGE:** Among apparently healthy adults, physiological markers that may reflect autonomic function—including an elevated resting heart rate, lower heart rate reserve, higher systolic blood pressure response to exercise, and slower heart rate recovery after exercise—were associated with a higher risk of cancer mortality, particularly lung cancer, independent of cardiorespiratory fitness. Awareness of these associations may help clinicians recognize these physiological responses as potential indicators of long-term cancer risk.

**TRANSLATIONAL OUTLOOK:** Our findings suggest a potential role of autonomic function in cancer development and progression and support further investigation of the autonomic nervous system in relation to cancer risk. Physiological measures reflective of autonomic regulation are increasingly accessible through consumer wearable devices, and if validated, they may support strategies for cancer prevention and early risk identification.

## Funding support and author disclosures

Dr Berry has received grant support from the 10.13039/100000002NIH, 10.13039/100016545Roche Diagnostics, and 10.13039/100014386Abbott Diagnostics; and consulting fees from The Cooper Institute, Roche Diagnostics, and AstraZeneca. Dr Gabriel has received grant support from the 10.13039/100000002NIH. All other authors have reported that they have no relationships relevant to the contents of this paper to disclose.
