# Supplementary material for: Markers of Autonomic Function and Cancer Mortality Risk in Healthy Adults
Source: JACC Adv. 2026 Sep 18;5(10):103242. doi: 10.1016/j.jacadv.2026.103242 (PMC13627117; doi:10.1016/j.jacadv.2026.103242)
Supplement: Supplementary file 1 [file mmc1.docx]

# Supplemental Table 1. Characteristics at clinic visit (1975-2018) among study participants in the HRR-1/2 subsample of the Cooper Center Longitudinal Study (n=43,022),^a^ overall and by sex and vital status at the end of follow-up

|  | Overall (n=43,022) | Men (n=29,348) | | Women (n=13,674) | |
| --- | --- | --- | --- | --- | --- |
|  |  | Cancer death  (n=532) | No cancer death  (n=28,816) | Cancer death  (n=190) | No cancer death  (n=13,484) |
| Characteristic |  | Mean (SD) | | | |
| Age (years) | 46.1 (9.6) | 53.8 (9.3) | 45.9 (9.2) | 54.0 (10.0) | 46.0 (10.2) |
| Body mass index (kg/m^2^) | 26.8 (4.7) | 28.4 (4.3) | 27.9 (4.3) | 25.6 (4.3) | 24.7 (4.8) |
| Current smoking^b^, n (%) | 5214 (12.3) | 100 (18.9) | 4315 (15.2) | 17 (9.1) | 782 (5.9) |
| Cardiorespiratory fitness (METs) | 10.8 (2.4) | 10.2 (2.4) | 11.4 (2.3) | 8.6 (1.9) | 9.6 (2.0) |
| Fasting glucose (mg/dL) | 97.5 (17.2) | 105.9 (26.2) | 99.6 (18.2) | 96.3 (12.5) | 92.7 (13.1) |
| Total cholesterol (mg/dL) | 198.5 (38.0) | 206.6 (39.6) | 199.1 (38.5) | 210.7 (36.3) | 196.8 (36.7) |
| Resting systolic blood pressure (mmHg) | 119.9 (14.1) | 126.3 (14.9) | 122.5 (13.0) | 119.0 (16.6) | 113.9 (14.3) |
| Resting diastolic blood pressure (mmHg) | 80.5 (10.0) | 83.6 (10.2) | 82.3 (9.6) | 78.3 (9.4) | 76.5 (9.5) |
| Heart rate recovery at 1-minute post-exercise (bpm)^c^ | 23.2 (8.2) | 19.9 (8.0) | 23.0 (8.0) | 20.6 (7.7) | 23.8 (8.7) |
| Heart rate recovery at 2-minutes post-exercise (bpm)^c^ | 42.6 (11.4) | 37.2 (11.9) | 42.5 (11.2) | 37.7 (11.0) | 43.2 (11.8) |

*Abbreviations: bpm, beats per minute; MET, metabolic equivalent; SD, standard deviation*

^a^The HRR-1/2 subsample included participants who had complete data on heart rate recovery at 1- and 2-minutes post-exercise.

^b^Percentages exclude missing observations. Current smoking was missing for 1.4% of men and 1.0% of women. Observations with missing smoking status were included in multivariable models using the maximum likelihood method.

^c^Heart rate recovery was calculated as: (maximal heart rate $-$ heart rate at 1- or 2-minutes post-exercise).

# Supplemental Table 2. Estimated hazard ratios for the association between heart rate recovery at 1- and 2-minutes and all-site cancer mortality among the HRR-1/2 subsample of the Cooper Center Longitudinal Study (n=43,022)

| Marker of autonomic function | Adjusted for age + lifestyle/clinical factors^a^ | Additional adjustment for CRF |
| --- | --- | --- |
| Heart rate recovery at 1-minute, per 10 bpm lower | 1.14 (1.04, 1.25) | 1.11 (1.01, 1.21) |
| Heart rate recovery at 2-minutes, per 10 bpm lower | 1.14 (1.07, 1.22) | 1.09 (1.02, 1.18) |

*Abbreviations: bpm, beats per minute; CI, confidence interval; CRF, cardiorespiratory fitness; HR, hazard ratio*

^a^Lifestyle and clinical factors included clinic visit age, exam year, sex, body mass index, smoking status, resting systolic blood pressure, fasting glucose, and total cholesterol.

# Supplemental Table 3. Estimated hazard ratios for the associations between markers of autonomic function and all-site cancer mortality among Cooper Center Longitudinal Study participants, excluding participants with less than five years of follow-up (n=70,399)

| Marker of autonomic function | HR (95% CI) | |
| --- | --- | --- |
| Men and Women (n=70,399) | Adjusted for age + lifestyle/clinical factors^a^ | Additional adjustment for CRF |
| Heart rate reserve, per 10% lower | 1.07 (1.04, 1.09) | 1.04 (1.02, 1.07) |
| Heart rate recovery at 5-minutes, per 10 bpm lower | 1.09 (1.06, 1.12) | 1.06 (1.03, 1.09) |
| Men (n=51,797)^b^ |  |  |
| Resting heart rate, per 10 bpm higher | 1.08 (1.04, 1.12) | 1.04 (1.00, 1.08) |
| Systolic blood pressure response, per 10 mmHg higher | 1.00 (0.98, 1.02) | 1.02 (1.00, 1.04) |
| Women (n=18,602)^b^ |  |  |
| Resting heart rate, per 10 bpm higher | 0.99 (0.91, 1.07) | 0.98 (0.90, 1.06) |
| Systolic blood pressure response, per 10 mmHg higher | 1.06 (1.02, 1.11) | 1.07 (1.03, 1.12) |

*Abbreviations: bpm, beats per minute; CI, confidence interval; CRF, cardiorespiratory fitness; HR, hazard ratio*

^a^Lifestyle and clinical factors included clinic visit age, exam year, sex, body mass index, smoking status, resting systolic blood pressure, fasting glucose, and total cholesterol.

^b^Models were sex-stratified when significant interactions were present.

# Supplemental Table 4. Estimated hazard ratios for the associations between markers of autonomic function and all-site cancer mortality among Cooper Center Longitudinal Study participants, excluding participants with a personal history of hypertension and those with an abnormal or equivocal resting ECG (n=60,662)

| Marker of autonomic function | HR (95% CI) | |
| --- | --- | --- |
| Men and Women (n=60,662) | Adjusted for age + lifestyle/clinical factors^a^ | Additional adjustment for CRF |
| Heart rate reserve, per 10% lower | 1.08 (1.05, 1.11) | 1.06 (1.03, 1.09) |
| Heart rate recovery at 5-minutes, per 10 bpm lower | 1.08 (1.05, 1.12) | 1.05 (1.02, 1.08) |
| Men (n=43,553)^b^ |  |  |
| Resting heart rate, per 10 bpm higher | 1.09 (1.05, 1.13) | 1.04 (1.00, 1.09) |
| Systolic blood pressure response, per 10 mmHg higher | 1.00 (0.98, 1.02) | 1.02 (1.00, 1.04) |
| Women (n=17,109)^b^ |  |  |
| Resting heart rate, per 10 bpm higher | 0.96 (0.88, 1.05) | 0.94 (0.86, 1.03) |
| Systolic blood pressure response, per 10 mmHg higher | 1.05 (1.01, 1.10) | 1.06 (1.01, 1.11) |

*Abbreviations: bpm, beats per minute; CI, confidence interval; CRF, cardiorespiratory fitness; ECG, electrocardiogram; HR, hazard ratio*

^a^Lifestyle and clinical factors included clinic visit age, exam year, sex, body mass index, smoking status, resting systolic blood pressure, fasting glucose, and total cholesterol.

^b^Models were sex-stratified when significant interactions were present.
